# Unusual non-nanophthalmic uveal effusion syndrome with histologically normal scleral architecture: a case report

**DOI:** 10.1186/s12886-020-01581-z

**Published:** 2020-07-29

**Authors:** Kasama Kaewsangthong, Somanus Thoongsuwan, Mongkol Uiprasertkul, Nopasak Phasukkijwatana

**Affiliations:** 1 Department of Ophthalmology, Faculty of Medicine Siriraj Hospital, Mahidol University, 2 Wanglang Road, Bangkoknoi, Bangkok, 10700 Thailand; 2 Department of Pathology, Faculty of Medicine Siriraj Hospital, Mahidol University, Bangkok, Thailand

**Keywords:** Choroidal hyperpermeability, Exudative retinal detachment, Nanophthalmos, Sclerectomy, Uveal effusion syndrome

## Abstract

**Background:**

To report an unusual case of non-nanophthalmic uveal effusion syndrome (UES) with histologically normal sclera but responsive to scleral resection.

**Case presentation:**

A73-year-old man presented with a bullous retinal detachment without ciliochoroidal detachment on funduscopic examination of the right eye. The axial length of both eyes was normal. Extensive investigations for possible causes of exudative retinal detachment were performed with unremarkable results except for choroidal hyperpermeability on indocyanine green angiography (ICGA). Ultrasound biomicroscopy (UBM) revealed scleral thickening with peripheral choroidal elevation leading to the diagnosis of UES.

Partial thickness sclerectomy and sclerotomy was performed resulting in complete retinal reattachment, reduction of choroidal hyperpermeability on ICGA and improvement of visual acuity. However, histological studies of the excised sclera revealed no scleral architectural changes or abnormal deposits.

**Conclusions:**

The diagnosis of UES in non-nanophthalmic eyes is challenging. Thorough systemic and ocular investigations are critical to rule out other etiologies. UBM can be helpful to evaluate scleral thickness and anterior choroid in equivocal cases. Our case was unique in that, although the sclera was thick, no abnormal microscopic scleral architecture could be identified. Misdiagnosis may lead to different surgical procedures such as vitrectomy resulting in unfavorable outcomes.

## Background

Idiopathic uveal effusion or uveal effusion syndrome (UES) is an extremely rare disease and often associated with nanophthalmic eyes [1]. The pathogenesis is thought to be related to abnormal sclera causing impaired scleral permeability and/or vortex vein compression, which leads to fluid accumulation in the choroid, ciliochoroidal detachment and accompanying exudative retinal detachment [2–4].

We reported a diagnostic challenging case of idiopathic UES in a non-nanophthalmic eye presented with retinal detachment without ciliochoroidal detachment seen on clinical examination. Our case was unique in that, despite the thickened sclera, histological studies revealed no scleral architectural changes and surgical management with scleral resection and sclerotomy was successful.

## Case presentation

A 73-year-old male presented with painless decreased visual acuity, floaters and flashes in the right eye for 2 weeks. The right eye did not have any previous history of trauma, surgery, cryotherapy or laser photocoagulation except for an uneventful cataract surgery 15 years ago. His left eye had undergone vitrectomy with retained silicone oil to treat retinal detachment for 8 months from another hospital. The left eye was now aphakic with retinal redetachment under the silicone oil, secondary glaucoma and band keratopathy. His medical underlying diseases were essential hypertension and hyperlipidemia. His systemic medications included amlodipine, losartan and simvastatin.

**Fig. 1:**
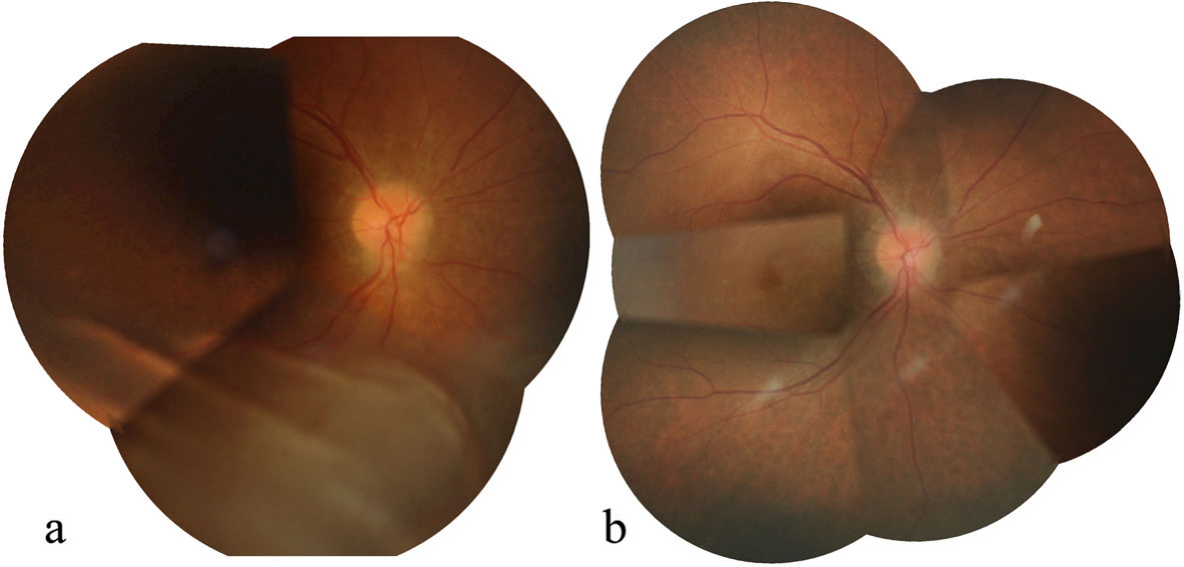
Color fundus photograph montage of the right eye illustrates corrugated inferior bullous retinal detachment at presentation (**a**) and complete retinal reattachment revealing a leopard spot pattern in the inferior fundus at 4 months after the surgery (**b**)

**Fig. 2:**
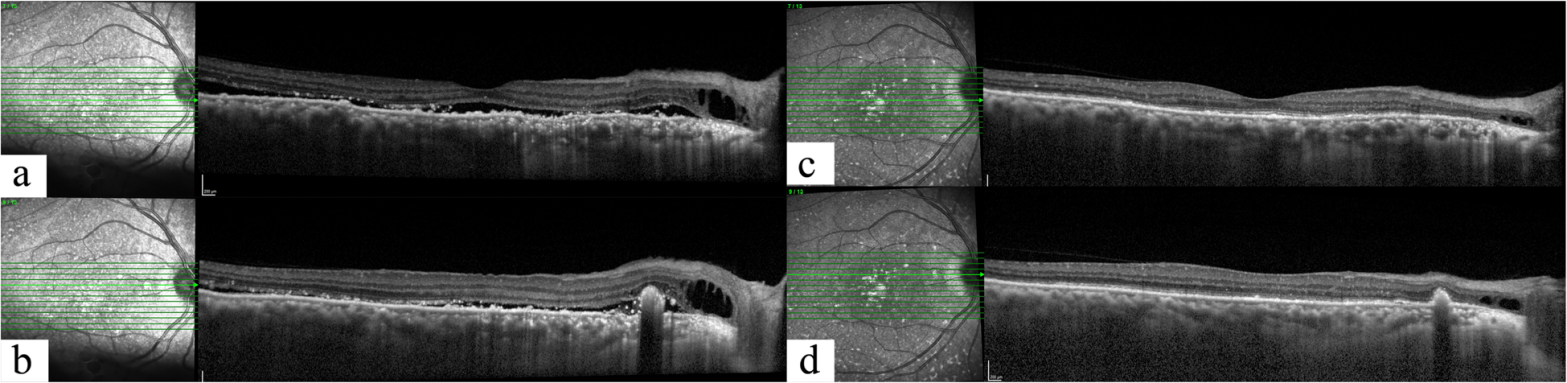
Optical coherence tomography of the macula of the right eye illustrates subretinal fluid, a small juxtapapillary pigment epithelial detachment and intraretinal cysts at presentation (**a** and **b**). At 4 months after surgery, the complete resolution of subretinal fluid was demonstrated (**c** and **d**)

At presentation, best-corrected visual acuity (BCVA) was 0.6 logMAR and counting fingers in the right and left eyes, respectively. Intraocular pressure (IOP) was 14 mmHg in the right eye and 12 mmHg in the left eye. Ocular examination of the right eye showed normal conjunctiva and sclera, clear cornea, mildly shallow anterior chamber with no cell or flare and the eye was pseudophakic. The pupil was dilatable to 5 mm. Dilated fundus examination revealed corrugated inferior bullous retinal detachment with shifting of subretinal fluid (Fig. 1a). No definite choroidal detachment or a retinal break was identified with a wide-field contact lens. Optical coherence tomography (SPECTRALIS, Heidelberg Engineering, Heidelberg, Germany) of the macula showed subretinal fluid, a small juxtapapillary pigment epithelial detachment and intraretinal cysts (Fig. 2a and b). At this point, rhegmatogenous retinal detachment with a very small retinal break versus exudative retinal detachment were to be differentiated. The patient was then worked up for possible etiologies of exudative retinal detachment including choroidal inflammation, choroidal tumors and atypical central serous chorioretinopathy.

**Fig. 3:**
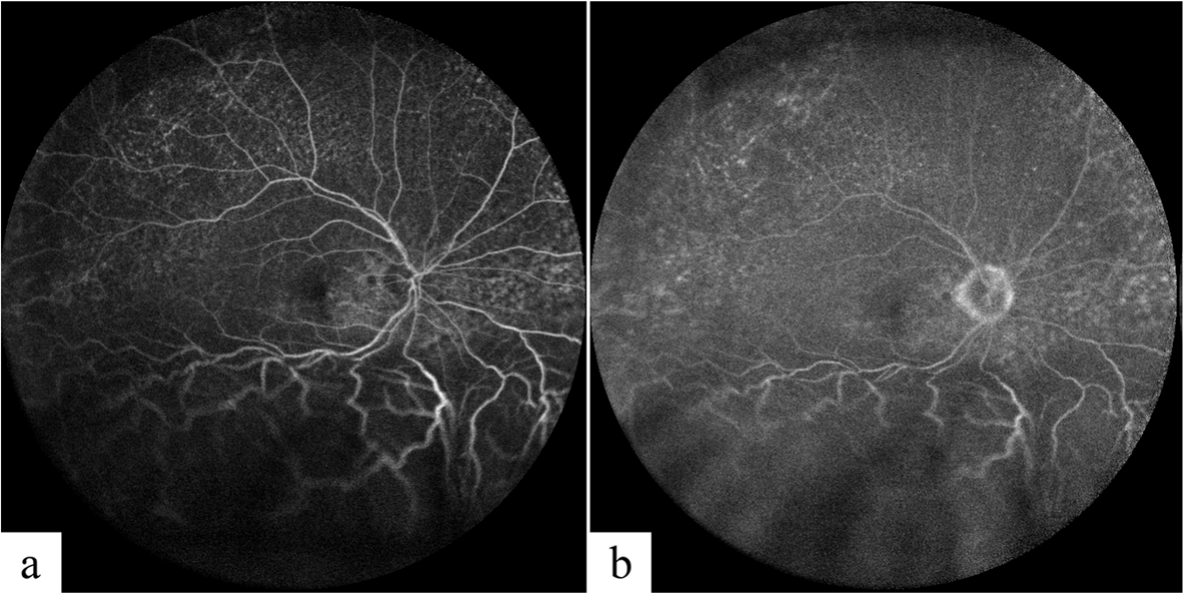
Fluorescein angiography of the right eye at presentation shows granular hyperfluorescence, consistent with window defects, at the mid-periphery and peripapillary areas at 1 min (**a**) and at 10 min (**b**). No dye leakage was detected to explain the subretinal fluid

**Fig. 4:**
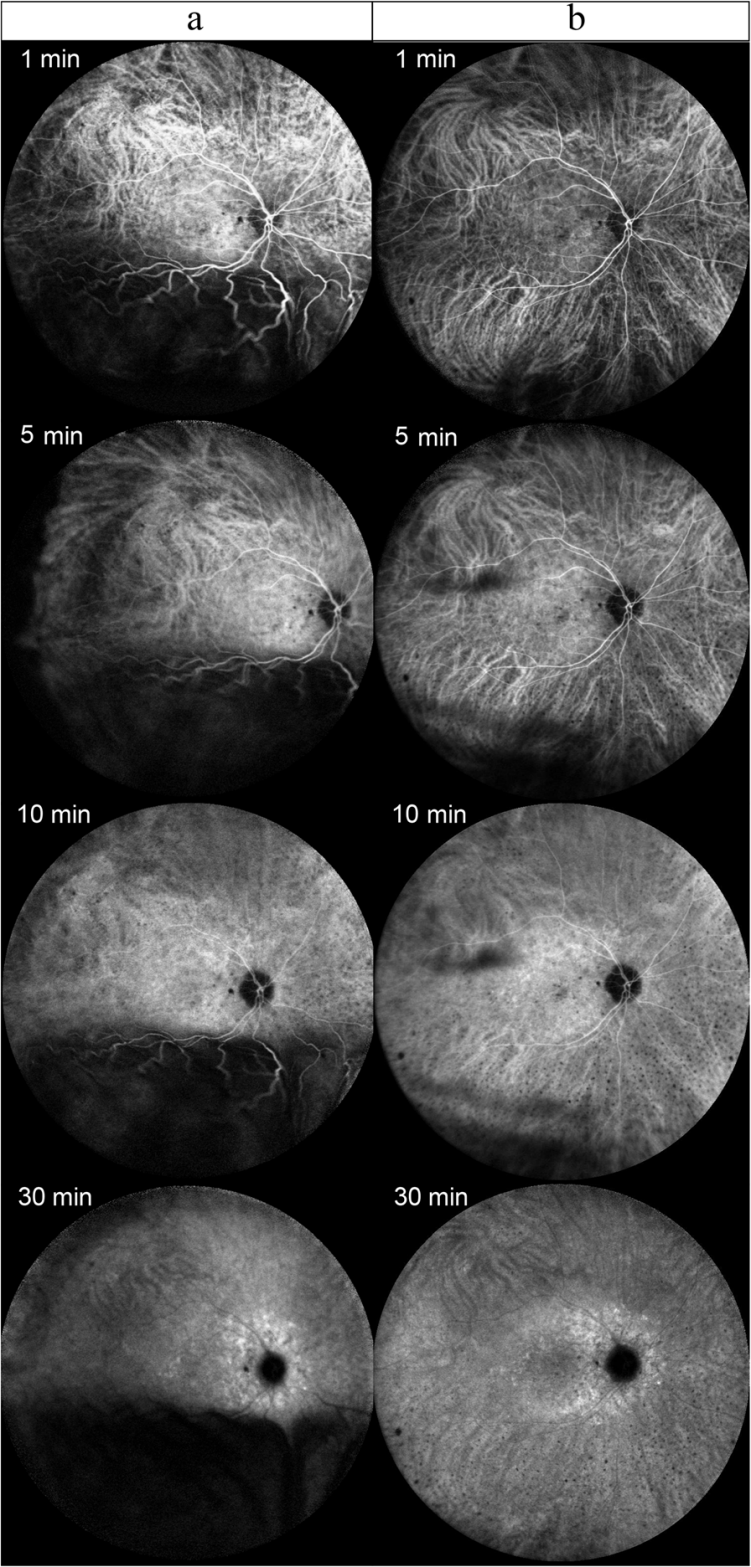
Indocyanine green angiography (ICGA) of the right eye. At presentation, ICGA demonstrates diffuse early hypercyanescence especially in the posterior pole and superotemporal vortex veins that persisted until the mid to late phase (10 min) and very late (30 min) hypercyanescence in the peripapillary area (Column **a**). One year after the surgery, follow-up ICGA reveals less choroidal hypercyanescence and the retina completely reattached (Column **b**)

**Fig. 5:**
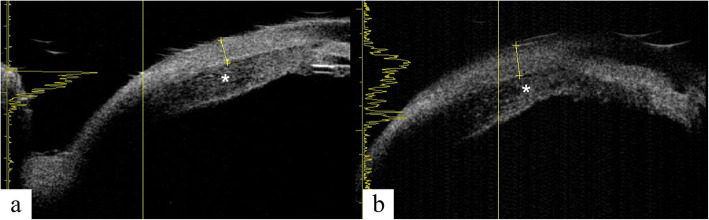
Ultrasound biomicroscopy of the right eye reveals edematous thickening of anterior choroid (**asterisk**) and thickening of the anterior sclera measuring 1.07 mm (**a**) and up to 1.26 mm (**b**)

Ocular B-scan ultrasonography (Aviso, Quantel Medical, Clermont-Ferrand, France) demonstrated ocular wall thickening with peripheral choroidal elevation in the right eye. No tumors were identified and no subtenon fluid collection to suggest posterior scleritis was detected. The axial length measurement (IOL Master 500, Carl Zeiss Meditec, Jena, Germany) was 22.59 and 22.94 mm in the right and left eyes, respectively, which was not compatible with nanophthalmos. Fluorescein angiography (FA) (SPECTRALIS, Heidelberg Engineering) showed granular hyperfluorescence, consistent with window defects, at the mid-periphery and peripapillary areas. No dye leakage was detected to explain the subretinal fluid, which made central serous chorioretinopathy unlikely (Fig. 3). Indocyanine green angiography (ICGA) (SPECTRALIS, Heidelberg Engineering) demonstrated diffuse early hypercyanescence especially in the posterior pole and superotemporal vortex veins that persisted to the late phase in the peripapillary area (Fig. 4, column a). FA and ICGA showed no evidence of chorioretinal inflammation, which made choroiditis such as sympathetic ophthalmia unlikely. Blood tests including complete blood count, blood chemistries, erythrocyte sedimentation rate, C-reactive protein, serological test for human immunodeficiency virus, venereal disease research laboratory test for syphilis, Treponema pallidum haemagglutination test, interferon gamma assay for tuberculosis, antinuclear antibodies test and antineutrophil cytoplasmic antibodies test were all within normal limit. The chest x-ray was also performed and showed no pulmonary infiltration. After excluding the infectious etiologies, 1 mg/kg/day of oral systemic steroid was challenged to the patient for 7 days without clinical responses. UES was now high in the differential diagnosis. The ultrasound biomicroscopy (UBM) (Aviso, Quantel Medical) was then performed and revealed edematous thickening of the anterior choroid and thickening of the anterior sclera measuring up to 1.26 mm (Fig. 5), leading to the diagnosis of UES (normal scleral thickness at the corneoscleral limbus is 0.53 ± 0.14 mm [5]).

Partial thickness sclerectomy and sclerotomy was then performed based on the technique described by Gass, 1983 [6]. In brief, two-thirds thickness scleral flaps of 5 mm × 7 mm were created centering at 2 mm anterior to the equator in each of the 4 quadrants. Full thickness sclerotomy (scleral window) of 2 mm × 2 mm was then made on each of the remaining scleral beds under the flaps. The scleral flaps from inferonasal and inferotemporal quadrants were excised and sent for histological examination.

**Fig. 6:**
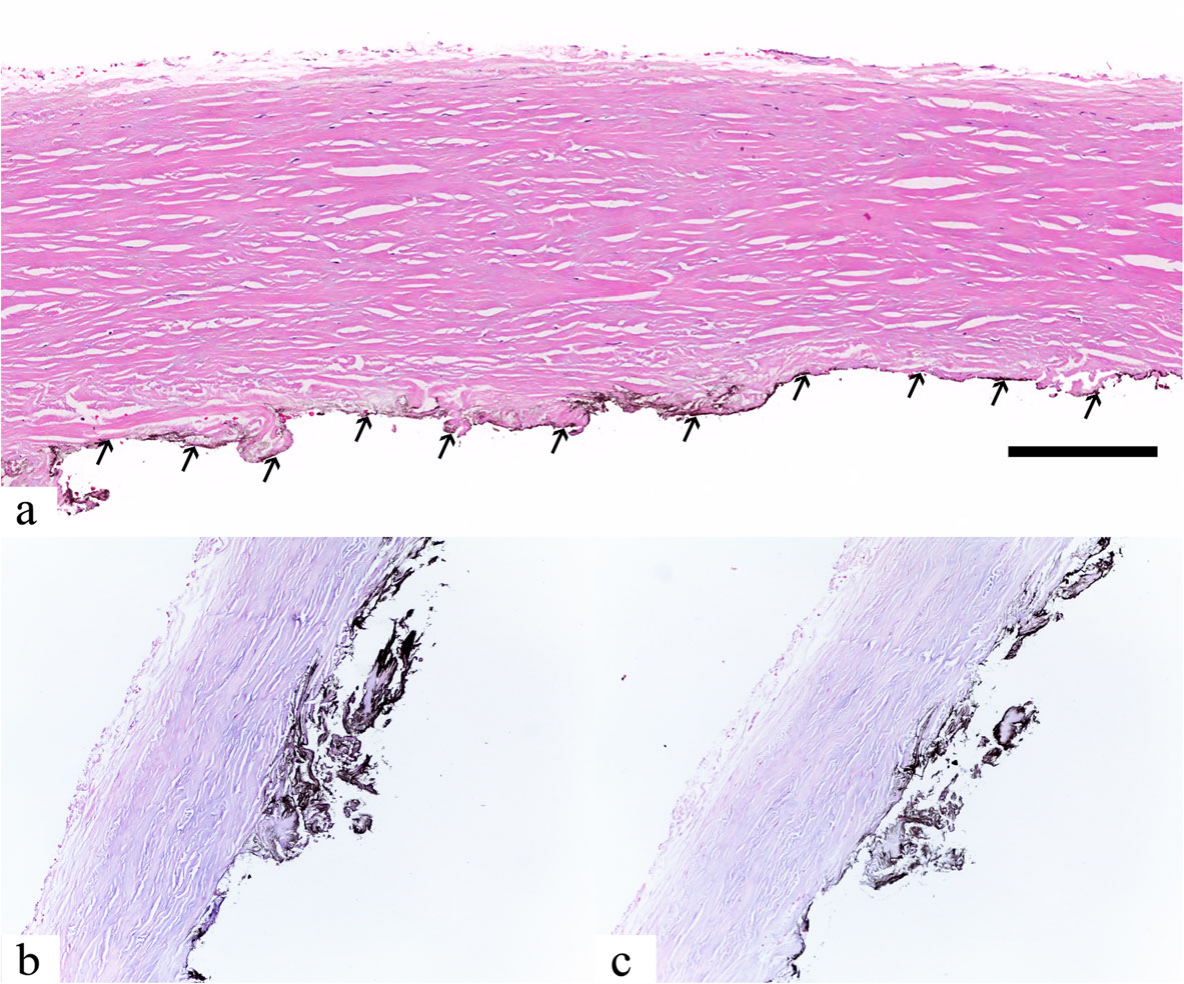
Histologic sections of the excised sclera show normal-appearing scleral architecture without inflammation or abnormal proteoglycans deposits. H&E staining (**a**), **arrows** indicated black ink. Bar = 200 μm. Alcian blue (**b**) and Alcian blue with hyaluronidase (**c**)

At 2-week post-operation, the BCVA improved to 0.2 logMAR in the right eye with the IOP of 16 mmHg and the subretinal fluid progressively reduced. At 4 months, the retina was completely attached, revealing a typical leopard spot pattern described in UES (Fig. 1b, 2c and d). One year after the surgery, follow-up ICGA revealed less choroidal hyperpermeability (Fig. 4, column b). Histologic findings (Fig. 6) of the excised sclera revealed neither inflammation nor disorganization of collagen fibers. Special stains including periodic acid–Schiff, periodic acid–Schiff–diastase, alcian blue, alcian blue with hyaluronidase, mucicarmine, congo red and reticulin showed no demonstrable proteoglycans or other abnormal substance deposits.

## Discussion and conclusions

We reported a diagnostic challenging case of UES presented with exudative retinal detachment without detectable ciliochoroidal detachment on clinical examination in a normal axial length eye. We demonstrated that UBM was a simple method to evaluate the scleral thickness and the anterior choroid to guide the diagnosis of UES in ambiguous cases. Our case was also unique in that there were no microscopic architectural changes of the sclera despite multiple histochemical stains, although the sclera was thickened and the surgical management with partial thickness sclerectomy with sclerotomy was successful.

Histologic studies of the sclera of UES demonstrated that increased scleral thickness was due to accumulation of proteoglycans, especially dermatan sulphate and chondroitin sulphate, causing resistance to transscleral flow and accumulation of fluid in the suprachoroidal and subretinal space [7, 8]. A comprehensive review by Uyama et al. [8] classified patients with UES into three types. Type 1 is nanophthalmic (axial length less than 19 mm) with high-graded hypermetropia and thickened sclera. Type 2 also has thick sclera, but with normal axial length and unremarkable refractive error. Histological studies of the excised sclera in types 1 and 2 revealed disorganized collagen fibers and deposition of proteoglycans. In contrast, type 3 has normal axial length, normal scleral thickness and normal scleral histology. Scleral resection and sclerotomy was an effective treatment only in type 1 and 2. According to this classification, our case was most compatible with type 2 but without histologically abnormal collagen fibers or proteoglycans deposits. Our case also suggested an alternative mechanism of increased scleral thickness not involving proteoglycans deposition of the sclera.

The pathogenesis of UES may involve several hypotheses including vortex vein compression, reduced scleral protein permeability, reduced scleral hydraulic conductivity, chronic hypotony and increased choroidal permeability [9]. Interestingly, ICGA in our case showed choroidal hyperpermeability seen as intense early hypercyanescence in the posterior pole that persisted till the late phase. The hyperpermeability became less in the follow-up ICGA at 1 year after the surgery with complete resolution of the subretinal fluid (Fig. 4, column b). This suggested that increased choroidal permeablility also play a significant role of uveal effusion in this patient. Interplay of multiple factors including relatively thick sclera and increased choroidal permeability might predispose this patient to UES. In addition, we noticed a peculiar finding of abnormal peripapillary hypercyanescence in the very late (30 min) ICGA. It might be possible that, apart from vortex veins, choroidal venous drainage in this patient occurred slowly through choroidopial veins in the peripapillary region [10] leading to the very late peripapillary hypercyanescence, although the exact mechanism of this peculiar finding remains unknown.

In summary, UES is a diagnosis of exclusion especially in cases of non-nanophthalmic eyes. Thorough systemic investigations and multimodal retinal imaging are critical to rule out other etiologies. UBM, a simple noninvasive investigation, can be very helpful to evaluate scleral thickness and anterior choroid in suspected UES cases. This unique case also illustrated UES with thick sclera but normal microscopic scleral architecture, which was unusual. Misdiagnosis may lead to different surgical procedures such as vitrectomy resulting in unfavorable outcomes, which might have had occurred in this patient’s fellow eye.

## Data Availability

All the data supporting our findings is contained within the manuscript. More data, in case of necessary, is available from the corresponding author on request.

## Abbreviations

BCVA: Best-corrected visual acuity
FA: Fluorescein angiography
ICGA: Indocyanine green angiography
IOP: Intraocular pressure
UBM: Ultrasound biomicroscopy
UES: Uveal effusion syndrome
